# Do financial constraint and perceived stress modify the effects of food tax schemes on food purchases: moderation analyses in a virtual supermarket experiment

**DOI:** 10.1017/S1368980024000077

**Published:** 2024-01-15

**Authors:** Sanne K Djojosoeparto, Maartje P Poelman, Michelle Eykelenboom, Mariëlle A Beenackers, Ingrid HM Steenhuis, Maartje M van Stralen, Margreet R Olthof, Carry M Renders, Frank J van Lenthe, Carlijn BM Kamphuis

**Affiliations:** 1 Department of Human Geography and Spatial Planning, Faculty of Geosciences, Utrecht University, Utrecht, The Netherlands; 2 Chair group Consumption and Healthy Lifestyles, Department of Social Sciences, Wageningen University & Research, Wageningen, The Netherlands; 3 Department of Health Sciences, Faculty of Science, Vrije Universiteit Amsterdam, and Amsterdam Public Health Research Institute, Amsterdam, The Netherlands; 4 Department of Public Health, Erasmus University Medical Center Rotterdam, Rotterdam, The Netherlands; 5 Department of Interdisciplinary Social Science, Faculty of Social and Behavioural Sciences, Utrecht University, Utrecht, The Netherlands

**Keywords:** Financial constraint, Perceived stress, Food-related taxes, Healthiness food purchases

## Abstract

**Objective:**

To investigate whether financial constraint and perceived stress modify the effects of food-related taxes on the healthiness of food purchases.

**Design:**

Moderation analyses were conducted with data from a trial where participants were randomly exposed to: a control condition with regular food prices, an sugar-sweetened beverage (SSB) tax condition with a two-tiered levy on the sugar content in SSB (5–8 g/100 ml: €0·21 per l and *≥*8 g/100 ml: €0·28 per l) or a nutrient profiling tax condition where products with Nutri-Score D or E were taxed at a 20 percent level. Outcome measures were overall healthiness of food purchases (%), energy content (kcal) and SSB purchases (litres). Effect modification was analysed by adding interaction terms between conditions and self-reported financial constraint or perceived stress in regression models. Outcomes for each combination of condition and level of effect modifier were visualised.

**Setting:**

Virtual supermarket.

**Participants:**

Dutch adults (*n* 386).

**Results:**

Financial constraint or perceived stress did not significantly modify the effects of food-related taxes on the outcomes. Descriptive analyses suggest that in the control condition, the overall healthiness of food purchases was lowest, and SSB purchases were highest among those with moderate/high levels of financial constraint. Compared with the control condition, in a nutrient profiling tax condition, the overall healthiness of food purchases was higher and SSB purchases were lower, especially among those with moderate/high levels of financial constraint. Such patterns were not observed for perceived stress.

**Conclusion:**

Further studies with larger samples are recommended to assess whether food-related taxes differentially affect food purchases of subgroups.

Substantial socioeconomic inequalities in obesity, other diet-related chronic diseases and dietary intakes exist, with higher prevalence rates of disease and unhealthier dietary patterns among people with a lower socioeconomic position^(1,2)^. Governmental food environment policies targeting the entire population, like sugar-sweetened beverage taxes (SSB taxes), are promising strategies to reduce obesity, diet-related chronic diseases and related inequalities in health and dietary intakes^(1,3–5)^. Such policies can be beneficial for overall population health, as these require little individual agency for behavioural change by creating an environment which stimulates healthy behaviour and discourages unhealthy behaviour^(3)^.

Over fifty countries worldwide, including eleven European countries, have already implemented SSB taxes, including Belgium, Finland, France, Hungary, Ireland, Latvia, Norway, Portugal, the UK, Poland, and Spain^(6,7)^. Evidence from countries in which an SSB tax has been implemented shows a decrease in SSB consumption^(8)^ or a lowering of sugar levels in SSB by producers (to avoid taxation)^(9)^. Taxation of a wide range of unhealthy foods and beverages instead of only SSB seems to have even more beneficial effects on diet quality and health^(10,11)^.

Some studies have shown that taxation may be more beneficial for dietary intakes of people in lower than people in higher socioeconomic groups^(12,13)^, although the evidence is inconclusive^(11,14)^. However, studies also reported concerns about the regressive burden of food-related taxes because unhealthy food consumption is associated with lower socioeconomic status^(15)^ and living on a small budget is more prevalent among lower socioeconomic groups, which makes the impact of these taxes larger for these groups^(16,17)^. To offset the regressive burden of food-related taxes and to prevent other potential unintended effects (e.g. increasing financial stress), combining taxation of unhealthy foods with price reductions of healthy foods, such as fruits and vegetables, may help^(17,18)^.

Potential mechanisms for the different socioeconomic effects of taxation might be related to the different material (e.g. income and housing) and psychosocial circumstances (e.g. social support) in which people in lower and higher socioeconomic groups are born, grow up, work, and age (i.e. daily living conditions)^(19,20)^. Unfavourable daily living conditions (e.g. low income and unemployment) to which people in lower socioeconomic groups are more often exposed than people in higher socioeconomic groups can lead to experiencing financial constraint^(21)^. This in turn may adversely influence healthy dietary behaviours^(19,21)^, as it is quite hard to eat a healthy and varied diet on a limited budget^(22)^. Indeed, studies have shown that experiencing financial constraint combined with the higher costs of healthy diets negatively influences people’s food choices and, with that, dietary quality^(23,24)^.

Unfavourable daily living conditions may also cause stress and worries, e.g. about inadequate housing conditions, potential job loss^(19,25)^. Further, less resources may be available to effectively cope with stressors (e.g. less social support and lower sense of control), which may make that demands quicker exceed the available resources, leading to higher levels of perceived stress^(26)^. Perceived stress may lead to unhealthier dietary behaviours as, explained in the scarcity theory, the energy and mental capacities needed to deal with stress leave less ‘cognitive bandwidth’ available to deal with other issues, like deliberately making healthy food choices^(27,28)^. Also, consuming unhealthy foods (e.g. snacking) can be used as a strategy to cope with perceived stress^(19,29,30)^.

Based on these reasonings, we arrive at two contrary hypotheses on how food-related taxes can have differential effects on people experiencing different levels of financial constraint and perceived stress. On the one hand, higher levels of financial constraint may make people more likely to pay close attention to prices of food products, prioritising low cost in food choices^(23)^. Indeed, studies have shown that people in low-income households are more price sensitive and as result are more likely to reduce their consumption in response to food-related taxes^(31)^. Thus, we hypothesise that people experiencing financial constraints are more likely to act upon price increases of unhealthy foods as a result of food taxation, and therefore more likely reduce unhealthy food consumption compared with people with no financial constraint. On the other hand, higher levels of perceived stress may lead to less cognitive bandwidth available for making deliberate food choices taking price increases into account^(27,28)^, especially of foods that are perceived as needed in order to cope with stress (e.g. SSB and snacks)^(29,30)^. Therefore, we hypothesise that people experiencing higher levels of perceived stress (are) less likely (able to) act upon price increases of unhealthy foods when food taxation is introduced, and therefore less likely reduce unhealthy food consumption compared with people with no perceived stress.

In a randomised controlled trial (RCT) in a virtual supermarket setting, we found that an SSB tax and nutrient profiling tax were effective in decreasing SSB purchases^(11)^. The nutrient profiling tax also increased the overall healthiness of food purchases and decreased the energy content^(11)^. The effects of an SSB tax and nutrient profiling tax on food purchases did not significantly differ across individuals with different educational levels^(11)^. Although the sample size of this RCT was relatively small (*n* 404) and subgroup analyses are subject to discussion^(32)^, the data collected with this RCT offer a unique opportunity to further explore two concrete, potential factors that may influence food purchases and could modify the effects of food-related taxes on the healthfulness of food purchases: financial constraint and perceived stress^(33)^. To explore theory-driven underlying mechanisms and what might work for whom, subgroup analyses are considered useful^(32)^. Thus, explorative analyses of trial data – even if underpowered – may provide useful first insights regarding formulated hypotheses and may show interesting patterns that should be later tested in more powered studies. Thus, such analyses of RCT data allowed us to investigate whether experiencing financial constraint or perceived stress may modify the effects of an SSB tax and a nutrient profiling tax on the healthfulness of food purchases.

## Methods

This study is a moderation analysis of an RCT that investigated the effects of an SSB tax and nutrient profiling tax on food purchases in a virtual supermarket setting^(11)^. More details about the methods, e.g. the recruitment of participants and procedures of the study can be found elsewhere^(11)^.

### Setting: The virtual supermarket

Data were collected in a Dutch virtual supermarket between June and August 2020^(34)^. A total of 580 food products were available in the Dutch virtual supermarket, including 119 types of non-alcoholic beverages. The Dutch Food Composition Database (NEVO) (online version 2019)^(35)^ was used to update the information on the nutritional composition of the products. Nutri-Scores were calculated using a calculation tool of the French National Public Health Agency^(36)^.

### Study design: a randomised controlled trial

Participants were randomly assigned to one of the following conditions in the virtual supermarket: (i) a control condition, (ii) an experimental condition with a two-tiered SSB tax or (iii) an experimental condition with a nutrient profiling tax:

- *Control condition (no tax).* In the control condition, regular prices were used. In the Netherlands, regular food prices include a value-added tax rate of 9 % that applies to all food and beverage products^(37)^. Moreover, a consumption tax of €0·0883 per l was applied to fruit and vegetable juices (including 100 % fruit and vegetable juices without added sugars), soft drinks and mineral water, with no distinction between SSB and sugar-free beverages (e.g. water or non-energetic sweetened beverages)^(38)^.
- *SSB tax condition.* In this condition, prices of SSB were taxed on a scheme similar to the UK Soft Drinks Industry Levy, applied to UK-produced or imported soft drinks containing added sugar^(4)^. Beverages containing 5–8 g of sugar per 100 ml are taxed €0·21 per l and beverages containing 8 g or more sugar per 100 ml are taxed €0·28 per l^(6)^. The levy does not apply to milk-based beverages, milk replacement beverages, alcohol replacement beverages, fruit juices without added sugar and powder used to make drinks^(6)^. In the virtual supermarket, the SSB tax rate corresponded to an average price increase of 22 percent for beverages liable for the levy. In total, 34 beverage products (6 percent of the stock of the virtual supermarket) were taxed.
- *Nutrient profiling tax condition.* In this condition, taxation of energy-dense, nutrient-poor foods and beverages was based on the Nutri-score. The Nutri-score is a nutrition label that presents the overall diet quality of foods and beverages on a five-point colour-coded scale from dark green (‘A’) to red (‘E’) using the British Food Standards Agency nutrient profiling system^(39)^. In this study, food and beverages with a label ‘D’ or ‘E’ were classified as ‘unhealthy (i.e. not contributing to a healthy diet)’ and taxed at a 20 percent level. In total, 225 foods and beverages (39 percent of the stock of the virtual supermarket) were taxed in this condition including 34 SSB.

To reflect a realistic situation in which the taxes were introduced^(40)^, participants in the experimental conditions were informed about the taxation before entering the virtual supermarket. Participants in the control condition did not receive such a notification.

### Recruitment of participants

Participants were invited by a research panel agency and were included if the following criteria were met: (i) being 18 years or older, (ii) being familiar with the Dutch language, (iii) being largely/totally responsible for grocery shopping in their household and (iv) having access to a laptop or computer. The RCT aimed to recruit a sample with an equal distribution of participants with a low, moderate and high educational level. In the first two months, less participants were recruited than expected, particularly among those with a low educational level. Therefore, additional efforts were undertaken to recruit participants (e.g. by means of additional reminders and an instruction video). Overall, 404 participants completed their shop in the virtual supermarket between June and August 2020. Participation was rewarded with panel member points that could be redeemed for cash (€4·00).

*Ethics of human subject participation:* The study was conducted according to the guidelines laid down in the Declaration of Helsinki. The trial protocol was evaluated by the Research Ethics Review Committee of the Faculty of Sciences, Vrije Universiteit Amsterdam. The trial protocol was registered in the Netherlands Trial Register (NTR) (registration number NL8616). All participants provided informed consent.

### Procedures

Participants were instructed to conduct a weekly grocery shop for their household (i.e. to buy the food and beverages they and the other members of their household need for a week) in the virtual supermarket. Participants were allocated a household-specific shopping budget, based on their household size and composition, according to the National Institute for Family Finance Information^(41)^. To illustrate, a two-adult-household received a shopping budget of 89 euros, whereas a household with two adults and two children in the age of 9–14 years received a shopping budget of 117 euros. When finished shopping, participants had to fill in an online closing questionnaire, to report on demographic characteristics (e.g. age, sex) and their living conditions (e.g. financial constraint and experienced stress).

### Measures

Three outcome measures were calculated based on the food and beverages participants had put in their shopping trolley during the shopping task in the virtual supermarket: (1) overall healthiness of the total weekly food shopping basket which was calculated as the % of food items with a Nutri-Score label ‘A’, ‘B’ or ‘C’ of the total weekly food shopping basket), (2) energy (kcal) content of the total weekly food shopping basket and (3) SSB purchases in litres in the total weekly food shopping basket. The overall healthiness and energy content of the total weekly food shopping basket followed a normal distribution. Because there was no normal distribution of residuals for SSB purchases and a large proportion of the participants did not purchase any SSB, SSB purchases were transformed into an ordinal variable, with the following categories: ‘0 l’ (reference category), ‘0–0·74 l’, ‘0·75–1·49 l’, ‘1·5–2·99 l’, ‘3–5·99 ’ and ‘6 l or more’.

#### Effect modifiers

##### Financial constraint

One item was included in the survey: ‘In the last 12 months, did you have difficulties making ends meet on your household income? ’ with answering options on a four-point Likert scale: (1) ‘No, no difficulties at all’, (2) ‘No, no difficulties, but I have to pay attention to my expenses’, (3) ‘Yes, some difficulties’ and (4) ‘Yes, many difficulties’. These last two categories were combined into one category ‘Yes, some or many difficulties’, because only a very small amount/percentage of the participants (15 out of the 394 included participants; 3·8 %) indicated to have many difficulties to make ends meet on their household income. This variable thus identifies three categories of financial constraint: (1) ‘no financial constraint’ (no difficulties at all), (2) ‘low level of financial constraint’ (having to pay attention to expenses) and (3) ‘moderate/high level of financial constraint’ (some or many difficulties). For the analyses, these categories were dummy coded, with ‘no financial constraint’ as the reference category.

##### Perceived stress

We used the four-item perceived stress scale developed by Cohen^(42)^ to assess the degree to which people feel that the demands in their lives exceed their abilities to cope effectively with these demands. Participants were asked four questions: (1) ‘In the past four weeks, how often have you felt that you were unable to control important things in your life?’; (2) ‘In the past four weeks, how often have you felt confident about your ability to handle personal problems?’; (3) ‘In the past four weeks, how often have you felt that things were going your way?’ and (4) ‘In the past four weeks, how often have you felt difficulties were piling up so high that you could not overcome them?’. Answers to each of these questions could be indicated on a five-point Likert scale ranging from 1 ‘always’ to 5 ‘never’. The items (1) and (4) were reverse coded, and based on the four items (Cronbach’s alpha’s 0·73), a mean score was calculated^(42)^, resulting in a continuous score that ranged from one to five, with higher scores representing higher perceived stress. In addition, the continuous variable perceived stress was mean centred by deducting the mean from the original variable.

#### Covariates

Despite the randomisation, there was some imbalance between the research conditions in the variables sex, educational level and BMI. Therefore, we included sex, educational level and BMI as covariates in the analyses. There was a small difference in household size between the research conditions. However, as this variable was proven to be a very strong predictor of the outcomes, we also included household size as a covariate in the analyses.

Household size was measured by summing up the number of people of different age categories (0–3 years; 4–8 years; 9–13 years and 14 years or older) living in households as reported by the participants. For sex, participants reported if they identified themselves as ‘female’, ‘male’ or ‘other’. Nine levels of education were distinguished: from (1) ‘no education’, (2) ‘lower education (primary school, special primary school)’, (3) ‘primary or pre-vocational education’, (4) ‘general secondary education’, (5) ‘secondary vocational education and apprenticeship training’, (6) ‘higher general secondary education and pre-university education (class 1–3)’, (7) ‘higher general secondary education and pre-university education (class 4–6)’, (8) ‘higher professional education’ and (9) ‘university education’. For the analyses, these educational levels were collapsed into three categories: (1) ‘low educational level’ (answers: 1–4, and 6), (2) ‘moderate educational level’ (answers: 5 and 7) and (3) ‘high educational level’ (answer 8 and 9)^(43)^, which were dummy coded, with ‘low educational level’ as the reference category. BMI was calculated using self-reported weight and height by participants (kg/m^2^). A BMI of <25 was considered a healthy weight, a BMI of 25 ≤ 30 as overweight and a BMI of ≥30 as obese^(44)^. The continuous covariate household size was centred around the median, and the continuous covariate BMI was centred around the mean by deducting the median or mean from the original variable.

#### Statistical analyses

Participants with extreme outliers (more than 3 * interquartile ranges below Q1 or above Q3) in any of the outcomes were excluded from all analyses (*n* 2). Moreover, participants who purchased only ≤5 different products in the virtual supermarket were excluded from the analyses (*n* 8), as this was considered implausible for a weekly grocery shop. Furthermore, we checked on missing values for self-reported financial constraint, perceived stress and the covariates (household size, sex, educational level and BMI). For BMI, we identified eight missing cases. Therefore, the final sample of our study included 386 participants. Descriptive statistics were reported using numbers, percentages, means and standard deviations (sd) or medians and interquartile ranges in case there was no normal distribution.

For the healthiness and energy content of food purchases, linear regression analyses were used to investigate whether effects of the SSB tax and nutrient profiling tax were different for individuals experiencing high or low levels of financial constraint or perceived stress. For SSB purchases, ordinal regression analysis was used.

We used the fully adjusted models as starting point of our analysis^(11)^. Separate models were run for each of the two effect modifiers (financial constraint and perceived stress) and for each of the three outcomes (overall healthiness, energy content and SSB purchases). We decided a prior to test for effect modification as well as to visualise the outcomes for each combination of condition and level of effect modifier, regardless of the statistically significance of the overall interaction term, since the sample size was relatively small and not powered for testing effect modification of our two potential modifiers. We first tested main effects, by adding experimental conditions and the effect modifier to the adjusted model (i.e. with the covariates household size, sex, educational level and BMI). Subsequently, effect modification was tested by adding the interaction terms between the conditions and the effect modifier to this model. We used the Generalised Linear Model function for the analysis of the overall interactions between the condition and the effect modifier on the three outcome measures and based assessment of significant interactions on the Wald *χ*^2^ test. We also assessed the separate interaction terms between the experimental conditions and three levels of financial constraint and between the experimental conditions and perceived stress for the three outcome measures, using linear regression models for the healthiness and energy content of food purchases and ordinal regression models for SSB purchases. All statistical tests were two sided. For each model, the effect sizes with corresponding 95 % CI and *P* values were computed. As the sample was relatively small and not powered on stratification, a significance level of *P* < 0·10 was chosen for effect modification^(45,46)^. Statistical analyses were performed using the software IBM SPSS Statistics 26·0. We also visualised the outcomes for each combination of condition and effect modifier, by summing up the constant value, effect of the condition and effect modifier and the statistical interaction effect. For perceived stress, we used the sd (–0·7 sd, Mean = 0, +0·7 sd) in the regression analyses. To visualise the outcomes for SSB purchases, we used the regression coefficients (B) for the calculations and converted the final outcomes to OR again.

## Results

### Participants

A slight majority of the participants was female (54·4 %), mean aged 48·4 years (sd 15·7) with a mean BMI of 26·7 (kg/m^2^) (sd 5·8) (Table 1). The mean household size was 2·3 persons (median 2). Sixty-four participants had a low educational level (16·6 %), and 80 (20·7 %) participants experienced a moderate/high level of financial constraint (Table 1). Participants had on average a score of 2·1 (sd 0·7) on the five point perceived stress scale, indicating almost never experiencing stress (Table 1).

**Table 1:** Descriptive statistics of the study participants, the potential modifying variables and consumer food purchases in the virtual supermarket

	Total (*n* 386)		Control condition (*n* 151)		SSBs tax condition (*n* 126)		Nutrient profiling tax condition (*n* 109)	
	*n* or mean	% or sd	*n* or mean	% or sd	*n* or mean	% or sd	*n* or mean	% or sd
Age (years), mean and sd	48·4	15·7	48·5	16·3	48·6	15·3	48·2	15·5
Sex, *n* and %								
Female	210	54·4	78	51·7	77	61·1	55	50·5
Male	176	45·6	73	48·3	49	38·9	54	49·5
Educational level, *n* and %								
Low	64	16·6	20	13·2	19	15·1	25	22·9
Moderate	141	36·5	44	29·1	56	44·4	41	37·6
High	181	46·9	87	57·6	51	40·5	43	39·4
BMI (kg/m^2^), mean and sd	26·7	5·8	27·5	6·0	26·5	5·7	26·0	5·4
Weight status, *n* and %								
Healthy weight (BMI < 25)	178	46·1	65	43·0	61	48·4	52	47·7
Overweight (BMI 25 ≤ 30)	128	33·2	49	32·5	38	30·2	41	37·6
Obese (BMI ≥ 30)	80	20·7	37	24·5	27	21·4	16	14·7
Household size, mean and sd	2·3	1·2	2·3	1·2	2·4	1·3	2·4	1·2
Household composition, mean and sd								
% of household 14 years or older	91·7	17·9	91·4	18·6	91·2	18·3	92·7	16·6
*Potential modifying variables*								
Stress								
Perceived stress*, mean and sd	2·1	0·7	2·1	0·7	2·2	0·8	2·1	0·8
Financial constraint								
Financial constraint†, *n* and %								
No financial constraint	159	41·2	61	40·4	59	46·8	39	35·8
Low level of financial constraint	147	38·1	58	38·4	42	33·3	47	43·1
Moderate/high level of financial constraint	80	20·7	32	21·2	25	19·8	23	21·1
*Consumer food purchases*								
SSB (litres), median and IQR	1·0	3·0	1·5	3·0	0·8	3·0	0·8	2·4
SSB, *n* and %								
0 l	171	44·3	58	38·4	60	47·6	53	48·6
0·75–1·5 l	29	7·5	10	6·6	9	7·1	10	9·2
1·5–3 l	83	21·5	39	25·8	23	18·3	21	19·3
3–6 l	74	19·2	30	19·9	26	20·6	18	16·5
6 l or more	29	7·5	14	9·3	8	6·3	7	6·4
Proportion healthy (%), mean and sd	71·5	10·9	70·8	10·2	71·6	11·1	72·4	11·6
Total energy (kcal), mean and sd	32 080	17 074	32 422	16 540	32 926	18 665	30 630	15 905

* Measured by four items: (1) ‘In the past four weeks, how often have you felt that you were unable to control the important things in your life?’; (2) ‘In the past four weeks, how often have you felt confident about your ability to handle your personal problems?’; (3) ‘In the past four weeks, how often have you felt that things were going your way?’ and (4) ‘In the past four weeks, how often have you felt difficulties were piling up so high that you could not overcome them?’, indicated on a five-point Likert scale from 1 ‘always’ to 5 ‘never’. The items (1) and (4) were reverse coded, and based on the four items, a mean score was calculated, resulting in a total continuous score that ranged from one to five, with higher scores representing higher experienced stress.

† Measured by one item ‘In the last 12 months, did you have difficulties making end meets on your household income?’ Indicated on a four-point Likert scale: (1) ‘No, no difficulties at all’, (2) ‘No, no difficulties, but I have to pay attention to my expenses’, (3)‘Yes, some difficulties’ and (4) ‘Yes, many difficulties’. These last two categories were recoded into one category, resulting in three levels of financial constraint: (1) ‘No financial constraint’, (2) ‘Low level of financial constraint’ and (3) ‘Moderate/high level of financial constraint’.

Participants bought on average 71·5 % healthy foods, 32 080 kcal and 1·0 l (median) of SSB in their total weekly shopping basket (Table 1). Less than half of the participants (44·3 %) purchased no SSB, and 7·5 % of the participants purchased 6 l of SSB or more (Table 1). In Supplemental File 1 more details can be found on the characteristics of the participants, their perceived stress, experienced financial constraint and consumer food purchases.

### Effect modification

Overall, we did not find statistically significant interactions between experiencing financial constraint and the experimental conditions for any of the three outcomes (overall healthiness of food purchases, Wald *X*^2^ = 4·54, *P* = 0·34; energy content, Wald *X*^2^ = 3·05, *P* = 0·55; SSB purchases, Wald *X*^2^ = 3·30, *P* = 0·51) (Model 2), nor between perceived stress and the experimental conditions for any of the outcomes (healthy food purchases, Wald *X*^2^ = 0·10, *P* = 0·95; energy content, Wald *X*^2^ = 0·70, *P* = 0·71; SSB purchases, Wald *X*^2^ = 0·07, *P* = 0·97) (Model 4). Further, no statistically significant interaction terms were found for the different combinations between the levels of financial constraint and the separate experimental conditions (Table 2). Similarly, there were no statistically significant interaction terms between perceived stress and the separate experimental conditions (Table 3).

**Table 2:** Effects of the experimental conditions, financial constraint and the experimental conditions*financial constraint on the three outcomes: the overall healthiness and energy content of the total weekly food shopping basket (linear regression analyses) and the likelihood of being in a lower-level category of sugar-sweetened beverage purchases (ordinal regression analyses)

	Overall healthiness (proportion healthy (%))						Energy content (kcal)						Likelihood of being in lower-level category of SSB purchases						
	Model 1a*			Model 2a†			Model 1b*			Model 2b†			Model 1c*			Model 2c†			
	*B*	95 % CI	*P*	*B*	95 % CI	*P*	*B*	95 % CI	*P*	*B*	95 % CI	*P*	OR	95 % CI	*P*	*B*	OR	95 % CI	*P*
Constant/threshold values	62·86	58·33, 67·39	<0·001	64·23	59·40, 69·05	<0·001	28 540·92	23 181·50, 33 900·34	<0·001	27 986·74	22 267·22, 33 706·25	<0·001	2·31 (0·75–1·49 l)	1·24, 4·33	0·009	0·76 (0·75–1·49 l)	2·13 (0·75–1·49 l)	1·08, 4·21	0·030
													1·64 (1·5–2·99 l)	0·88, 3·07	0·120	0·41 (1·5–2·99 l)	1·51 (1·5–2·99 l)	0·77, 2·98	0·234
													0·56 (3–5·9 l)	0·30, 1·04	0·070	−0·67 (3–5·9 l)	0·51 (3–5·9 l)	0·26, 1·01	0·055
													0·11 (≥ 6 l)	0·05, 0·21	<0·001	−2·33 (≥ 6 l)	0·10 (≥ 6 l)	0·05, 0·20	<0·001
Control condition	Reference			Reference			Reference			Reference			Reference				Reference		
SSB tax condition	1·26	−1·27, 3·80	0·328	−1·10	−4·88, 2·68	0·567	−1350·36	−4347·25, 1646·53	0·376	630·94	−3853·28, 5115·15	0·782	1·62	1·16, 3·02	0·037	0·33	1·39	0·71, 2·74	0·342
Nutrient profiling condition	2·74	0·10, 5·38	0·042	0·11	−4·15, 4·37	0·960	−3396·59	−6517·77, –275·40	0·033	−3055·41	−8103·04, 1992·22	0·235	1·87	1·03, 2·55	0·010	0·41	1·51	0·71, 3·22	0·288
No financial constraint	Reference			Reference			Reference			Reference			Reference				Reference		
Low level of financial constraint	−.78	−3·16, 1·59	0·517	−3·28	−7·07, 0·50	0·088	2215·77	−591·34, 5022·87	0·121	4507·75	25·24, 8990·26	0·049	1·04	0·68, 1·60	0·856	−0·13	0·88	0·45, 1·71	0·700
Moderate/high level of financial constraint	−1·83	−4·68, 1·03	0·209	−4·56	−9·08, –0·05	0·047	−371·04	−3744·84, 3002·76	0·829	−578·40	−5927·80, 4771·00	0·832	1·02	0·62, 1·70	0·929	−0·19	0·83	0·38, 1·82	0·635
Control condition x no financial constraint	Reference			Reference			Reference			Reference			Reference				Reference		
Low level of financial constraint* Nutrient-profiling tax condition	–	–	–	3·67	−2·20, 9·54	0·220	–	–	–	−2201·76	−9160·36, 4756·84	0·534	–	–	–	0·15	1·16	0·40, 3·31	0·787
Moderate/high level of financial constraint* Nutrient profiling tax condition	–	–	–	5·69	−1·36, 12·74	0·113	–	–	–	2088·50	−6266·77, 10 443·76	0·623	–	–	–	0·85	2·33	0·62, 8·63	0·205
Low level of financial constraint * SSB tax condition	–	–	–	4·60	−1·04, 10·24	0·110	–	–	–	−5124·00	−11 808·01, 1560·01	0·133	–	–	–	0·45	1·57	0·56, 4·39	0·387
Moderate/high level of financial constraint * SSB tax condition	–	–	–	3·43	−3·24, 10·09	0·313	–	–	–	−1045·59	−8948·22, 6857·04	0·795	–	–	–	−0·02	0·98	0·30, 3·18	0·977

* Main effects of experimental conditions and financial constraint, controlled/adjusted for household size, sex (male is reference), educational level (low educational level is reference) and BMI.

† Effects of experimental conditions (in the group with no financial constraint) and effects of financial constraint (in the control condition), controlled/adjusted for household size, sex (male is reference), educational level (low educational level is reference), BMI, with interaction terms between the experimental conditions and financial constraint.

**Table 3:** Effects of the experimental conditions, perceived stress and the experimental conditions*perceived stress on the three outcomes: the overall healthiness and energy content of the total weekly food shopping basket (linear regression analyses) and on the likelihood of being in a lower-level category of sugar-sweetened beverage purchases using ordinal regression analyses (ordinal regression analyses)

	Overall healthiness (proportion healthy (%))						Energy content (kcal)						Being in a lower-level category of SSB purchases						
	Model 3a*			Model 4a†			Model 3b*			Model 4b†			Model 3c*			Model 4c†			
	*B*	95 % CI	*P*	*B*	95 % CI	*P*	*B*	95 % CI	*P*	*B*	95 % CI	*P*	OR	95 % CI	*P*	*B*	OR	95 % CI	*P*
Constant/threshold values	61·98	57·62, 66·35	<0·001	62·02	57·64, 66·41	<0·001	29 279·12	24 085·58, 34 472·67	<0·001	29 401·22	24 189·80, 34 612·65	<0·001	2·39 (0·75–1·49 l)	1·33, 4·29	0·004	0·88 (0·75–1·49 l)	2·41 (0·75–1·49 l)	1·34, 4·35	0·003
													1·69 (1·5–2·99 l)	0·94, 3·03	0·077	0·54 (1·5–2·99 l)	1·71 (1·5–2·99 l)	0·95, 3·07	0·074
													0·57 (3–5·9 l)	0·32, 1·02	0·058	−0·56 (3–5·9 l)	0·57 (3–5·9 l)	0·32, 1·03	0·064
													0·11 (≥ 6 l)	0·06, 0·21	<0·001	−2·22 (≥ 6 l)	0·11 (≥ 6 l)	0·06, 0·21	<0·001
Control condition	Reference			Reference			Reference			Reference			Reference				Reference		
SSB tax condition	1·37	−1·15, 3·89	0·285	1·71	−6·11, 9·54	0·667	−1438·77	−4433·07, 22 449·23	0·345	−374·33	−9671·35, 8922·69	0·937	1·65	1·05, 2·59	0·032	0·45	1·56	0·39, 6·33	0·534
Nutrient profiling tax condition	2·73	0·10, 5·36	0·042	1·95	−5·91, 9·80	0·626	−3288·30	−6412·35, –164·25	0·039	−5836·42	−15 164·92, 3492·09	0·219	1·86	1·16, 2·30	0·010	0·74	2·11	0·52, 8·58	0·299
Perceived stress	−1·20	−2·60, 0·21	0·095	−1·26	−3·68, 1·16	0·305	−870·44	−2536·82, 795·94	0·305	−1099·29	−3971·56, 1772·98	0·452	0·75	0·59, 0·97	0·025	−0·28	0·76	0·50, 1·16	0·200
Control condition x perceived stress	Reference			Reference			Reference			Reference			Reference				Reference		
Perceived stress * Nutrient profiling tax condition	–	–	–	0·36	−3·08, 3·81	0·835	–	–	–	1186·84	−2900·27, 5273,95	0·568	–	–	–	−0,06	0·95	0·51, 1·74	0·856
Perceived stress * SSB tax condition	–	–	–	−0·16	−3·59, 3·27	0·928	–	–	–	−493·40	−4568·06, 3581·27	0·812	–	–	–	0,02	1·02	0·56, 1·87	0·938

* Main effects of experimental conditions and perceived stress, controlled/adjusted for household size, sex (male is reference), educational level (low educational level is reference) and BMI.

† Effects of experimental conditions (in the group with no perceived stress) and perceived stress (in the control condition), controlled/adjusted for household size, sex (male is reference), educational level (low educational level is reference) and BMI, with interaction terms between the experimental conditions and perceived stress.

**Fig. 1:**
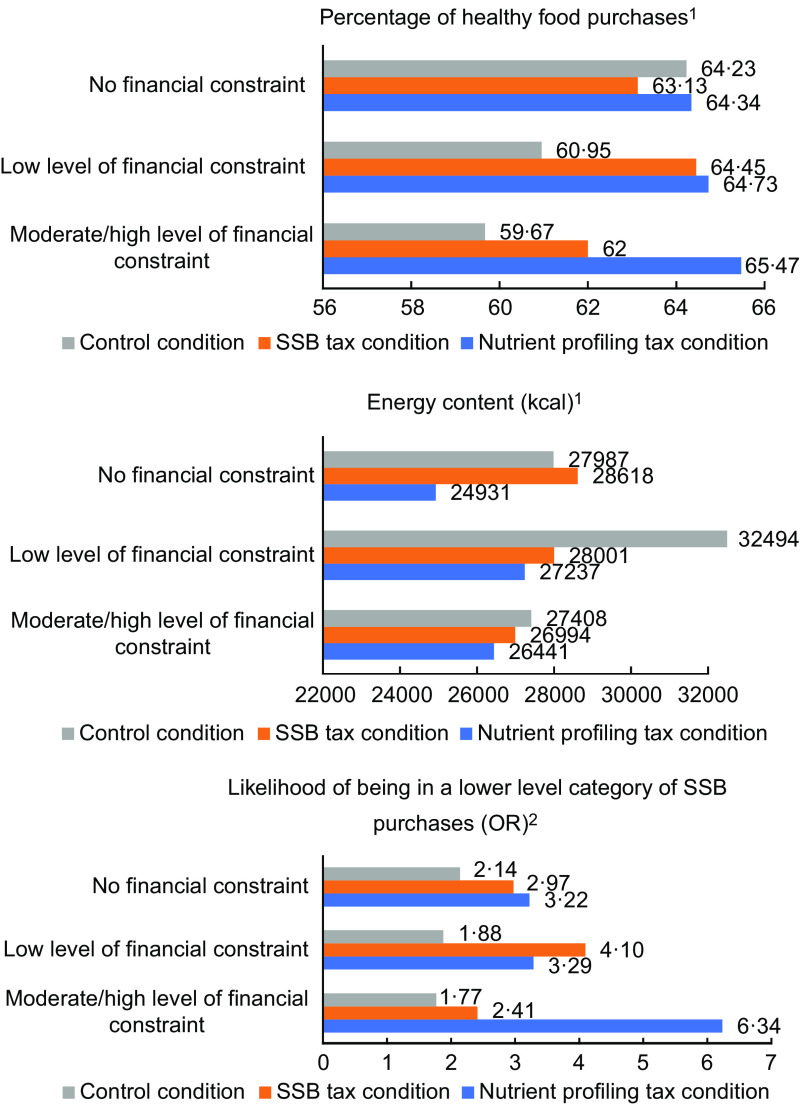
The percentage of healthy food purchases, energy content and likelihood of being in a lower level of SSB purchases visualised for each combination of level of financial constraint and condition, for men, with a low educational level, median household size and mean BMI. ^1^Calculated by summing up the constant value (B), effect of the condition, effect of the level of financial constraint and the interaction term of condition*level of financial constraint. ^2^Calculated by summing up the constant value (B), effect of the condition, effect of the level of financial constraint and the interaction term of condition*level of financial constraint. Final outcomes were converted to ORs again. Calculations in this figure are based on the constant value (B) of the category 0·75–1·49 l SSB purchases.

**Fig. 2:**
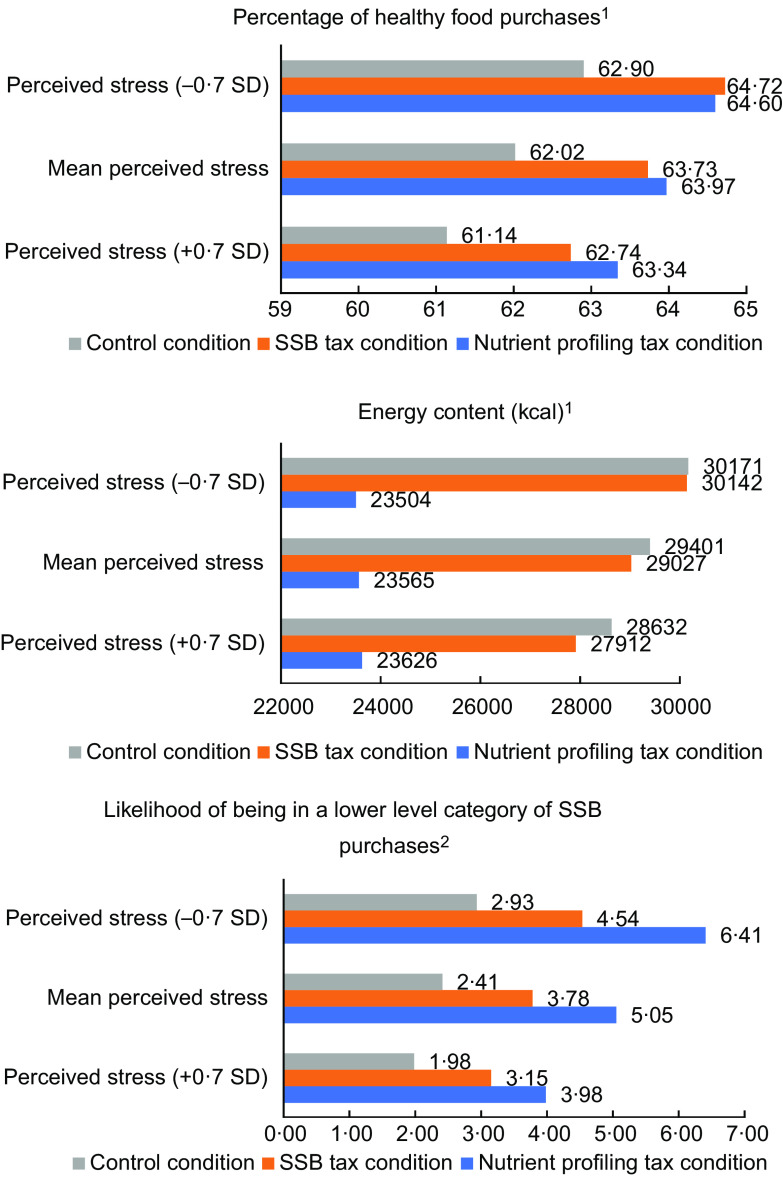
The percentage of healthy food purchases, energy content and likelihood of being in a lower level of SSB purchases visualised for each combination of perceived stress (–0·7 sd, Mean = 0, +0·7 sd) and condition, for men, with a low educational level, median household size and mean BMI. ^1^Calculated by summing up the constant value (B), effect of the condition, effect of perceived stress and the interaction term of condition*perceived stress. ^2^Calculated by summing up the constant value (B), effect of the condition, effect of perceived stress and the interaction term of condition*perceived stress. Final outcomes were converted to ORs again. Calculations in this figure are based on the constant value (B) of the category 0·75–1·49 l SSB purchases. *For perceived stress, we used the sd (–0·7 sd, Mean = 0, +0·7 sd) in the regression analyses.

The percentage of healthy food purchases, energy content and likelihood of being in a lower level of SSB purchases have been visualised for each combination of level of financial constraint and condition in Fig.1. In this figure, we observed differential patterns on the percentage of healthy food purchases and the likelihood of being in a lower-level category of SSB purchases for people experiencing different levels of financial constraint. So can be observed that in the control condition, the percentage of healthy food purchases and the likelihood of being in a lower-level category of SSB purchases is lower among people experiencing low or moderate/high levels of financial constraint compared with people experiencing no financial constraint. Compared with the control condition, the percentage of healthy food purchases and the likelihood of being in a lower-level category of SSB purchases is higher in the nutrient profiling tax condition, especially among people with moderate to high levels of financial constraint. Also, we observe a higher percentage of healthy food purchases and a higher likelihood of being in a lower-level category of SSB purchases among people experiencing moderate/high levels of financial constraint compared with people experiencing no financial constraint in the nutrient profiling condition. For the SSB tax, we observe similar patterns among people experiencing low levels of financial constraint, although effects are smaller than in the nutrient profiling tax condition in the highest financial constraint group. We did not observe any differential patterns for the third outcome (energy content of food purchases) (Fig. 1). For perceived stress, we observed a higher percentage of healthy food purchases, a higher likelihood of being in a lower-level category of SSB purchases and less energy content among all subgroups in the nutrient profiling tax condition as well as in the SSBs tax condition compared with the control condition. However, we did not observe any differential effects of these taxes, but similar patterns on food purchases for people with lower and higher levels of perceived stress (Fig. 2).

## Discussion

We did not find evidence for a modifying role of financial constraint or perceived stress on the effect of any of the experimental conditions on the healthfulness of food purchases. Therefore, the results did not confirm our hypotheses that people experiencing financial constraint are more likely to act upon price increases of unhealthy foods as a result of food-related taxes, nor that people experiencing higher levels of perceived stress are less likely to act upon price increases as a result of food-related taxes compared with people with no financial constraint or perceived stress.

We did not come across other studies which investigated the modifying effect of experiencing financial constraint or perceived stress on the relation between food-related taxes and food purchases. However, several studies showed that price increases and taxes on specific (e.g. SSB, high energy dense) foods have a greater impact on people with a lower socioeconomic position^(12)^, as they are more price-sensitive and as a result reduce purchases of these foods more than people with a higher socioeconomic position^(12,47,48)^. However, another study found that the effects of a 25 % price increase on unhealthy products alone on the percentage of healthy food purchases were not modified by income level, but that if these price changes were also communicated and combined with nudging strategies, there was a small significant increase in healthy food purchases in low-income groups, while for high-income participants, no statistically significant increase was found^(14)^.

That we found no statistical evidence for a modifying role of financial constraint or perceived stress on the effect of any of the experimental conditions on the healthfulness of food purchases might be explained by a lack of power in our study sample to detect statistical interactions. A total of 81 study participants experienced a moderate/high level of financial constraint, and the mean score on perceived stress in this study was 2·1, which is corresponding with the answer category ‘almost never’ perceiving stress. It might be that this sample included relatively few people experiencing higher levels of stress compared with people experiencing no perceived stress, also because the odds of participating in an experiment may be higher for those not experiencing stress^(28)^. Although often a sample is not powered for a secondary analysis into effect modification (like in our case), it is still important to carry out such theory-based, modification analyses in order to gain insight in potentially differential effects of interventions among population subgroups and thereby uncover potential pro-equity effects of interventions^(32)^.

However, based on the visualisations of the effects of financial constraint and the conditions on the overall healthiness of food purchases and SSB purchases, we did make some noteworthy observations. These observations suggest that in a situation without taxes, people experiencing moderate to high levels financial constraint purchase less healthy food purchases compared with people experiencing no financial constraint. The observations also suggest that compared with the control condition, in a nutrient profiling tax condition, the overall healthiness of food purchases was higher, and SSB purchases were lower especially among people experiencing moderate/high levels of financial constraint, more than among people with no financial constraint, which would be in line with our first hypothesis. The larger impact of food-related taxes on people experiencing higher levels of financial constraint may be considered unfair for people with already often smaller budgets^(49)^. However, food-related taxes can also have progressive health effects when especially people with lower incomes (which can lead to experiencing higher levels of financial constraint), as a result of price increases on unhealthy foods, substantially reduce consumption of these foods^(17)^. This is also in line with findings of a qualitative study in the Netherlands, in which stakeholders of various organisations (e.g. health professional and health consumer organisations, academia, trade associations, ministries and parliamentary parties) expected an SSB tax to have a financially regressive effect, but would therefore also have the potential to especially reduce the SSB consumption among people with lower incomes^(18)^, which could reduce socioeconomic inequalities in diet and health. Nevertheless, the review of Wright et al. including empirical studies states that available research does not sufficiently address the question of whether the progressive effects exceed the regressive effects of health taxes^(17)^. However, a study that analysed the ethical implications of SSB taxation suggests that there is a strong ethical case for food-related taxes that likely promote greater equality because the largest health benefits from the tax are expected to be accrued to lower socioeconomic groups, even more so when revenues are spent on health and social equity^(49)^.

We did not observe differential patterns between subgroups on the effects of food-related taxes for the third outcome (energy content). In the main study, the energy content was lower (–3301 kcal) for participants in the nutrient profiling tax condition than for those in the control condition^(11)^. However, no significant effect of the SSB tax on the energy content of the total weekly shopping basket was observed^(11)^. A likely explanation for this might be that SSB purchases account for only a small part of total food purchases, which makes it less likely that a significant effect will be detected with the included sample size of this study^(11)^. Further, as a result of an SSB tax, participants may also have substituted SSB with other high-energy products^(11)^, although studies into SSB substitution patterns have been inconclusive^(31,50,51)^.

Furthermore, we did not find clear differential patterns between those with lower or higher levels of perceived stress for any of the three outcomes. Further research is needed to investigate whether effects of food taxation are more pronounced among those experiencing higher levels of financial constraint or perceived stress.

### Strengths and limitations

An important strength of this study is that this is, as far as we are aware, the first study that investigated the modifying effects of experiencing financial constraint and perceived stress on the effects of food-related taxes on food purchases. Another strength is that next to the effects of an SSB tax, this study also included the taxation of a wider range of unhealthy foods by using the nutrient profiling tax, which even seem to have more beneficial effects on overall diet quality and health^(10,11)^.

The main limitation, as discussed earlier, is the small sample and the lack of statistical power to conduct the moderation analyses. Another limitation might be the use of a virtual supermarket that is not identical to a real-life supermarket^(33)^. For instance, participants do not spend real money, and the allocated shopping budget was based on household composition and size but not on actual income levels, which may have influenced their shopping behaviour, especially of participants experiencing financial constraint, by paying less attention to prices than they do in real life. Further, reminding people of taxation on unhealthy foods and drinks just before entering the supermarket might have led people who otherwise would not have paid much attention to public health messages, to pay more attention to prices in the virtual supermarket. Furthermore, although studies have shown that in general shopping patterns in a virtual supermarket resemble those in real life^(14)^, it is unknown how shopping patterns would differ for subgroups experiencing different levels of financial constraint or perceived stress. Nevertheless, the use of a 3D virtual supermarket might also be seen as a strength, resembling a real store and given the much higher costs and logistic difficulties attached to conducting research in physical stores, as well as the fact that online grocery shopping is becoming increasingly common. However, the lay out of the 3D virtual supermarket used in this study differs from current online supermarkets (e.g. less elaborate and less convenient).

Furthermore, limitations might be that financial constraint was measured by one time and that different time periods were taken into account for measuring financial constraint (12 months) and perceived stress (1 month). However, the Perceived Stress Scale is one of the most widely disseminated methods of assessing psychological stress, and the four-item Perceived Stress Scale has also proven to be a useful instrument for assessing stress perception levels in the general population in different countries^(52)^.

Finally, the RCT was conducted in times of the COVID-19 pandemic, which might have influenced the state of mind of people, because in media it was reported that people with overweight had a higher risk of severe disease when infected with the Corona virus. However, the RCT found that a majority of the participants (82 %) reported to not have changed their food purchases due to COVID-19, suggesting that conditions surrounding the COVID-19 pandemic did not have a major effect on our findings^(11)^.

### Implications for practice and suggestions for future research

We believe that there are reasons warranting further investigations to assess whether food-related taxes differentially affect subgroups with different material and psychosocial circumstances, such as financial constraint and perceived stress. This is especially relevant in current insecure times with high inflation rates and high energy prices^(53)^, with as a result an increasing number of people having difficulties making ends meet and experiencing stress. Studies could assess whether the overall health benefits of food-related taxes will exceed the financially regressive effects of taxation^(17)^, or which other potential negative side effects could be caused by taxation (e.g. financial stress). We recommend using hypotheses in these studies based on theories that take elements of broader daily living conditions into account, as dietary and health inequalities are caused by a complex set of interrelated factors (e.g. food environment exposures, living conditions and individual-level factors)^(20,54)^.

These studies could be used to strengthen the evidence base and inform policymakers on how to effectively implement food-related taxes, aiming to improve population diets in general but also specifically targeting the most vulnerable (e.g. lower socioeconomic, lower income) groups, without posing unnecessary burdens on them. In order to be effective and prevent food-related taxes from increasing dietary and budgetary inequalities, it is recommended to combine these kinds of price interventions with other food environment policies^(18)^ (e.g. decreasing the prices of healthy foods, providing fruit and vegetable subsidies targeting lower income populations^(55)^), but also with policies tackling more distal determinants of unhealthy diets (e.g. financial debts, deprived housing conditions and social problems)^(54)^. The application of a systems perspective (a system of multiple, interconnected factors exerting non-linear influence on dietary intakes) can enhance the development of effective policies tackling dietary and health inequalities, while also shining a light on the potential unintended consequences^(56)^.

## Conclusions

Our study did not provide evidence that the effects of an SSB tax or nutrient profiling tax on the healthfulness of food purchases were modified by experiencing different levels of financial constraint or perceived stress. Future studies with larger samples, using theory-based hypotheses that take elements of broader daily living conditions into account, are recommended to assess whether food-related taxes differentially affect the healthfulness of dietary intakes of subgroups of the population. These studies could be used to strengthen the evidence base and inform policymakers on how to effectively implement food-related taxes, aimed at improving dietary intake in populations and specifically targeting the most vulnerable groups, without posing unnecessary burdens on them.

## Supporting information

Djojosoeparto et al. supplementary material Djojosoeparto et al. supplementary material

## Supplementary material

For supplementary material accompanying this paper visit https://doi.org/10.1017/S1368980024000077
