# Supplementary material for: Do financial constraint and perceived stress modify the effects of food tax schemes on food purchases: moderation analyses in a virtual supermarket experiment
Source: Public Health Nutr. 2024 Jan 15;27(1):e38. doi: 10.1017/S1368980024000077 (PMC10897579; doi:10.1017/S1368980024000077)
Supplement: Djojosoeparto et al. supplementary material [file S1368980024000077sup001.docx]

**Supplementary file 1**

**Table S1.** Descriptive statistics of the study participants, the potential modifying variables and consumer food purchases in the virtual supermarket.

|  | Total (*n* 386) | | Control condition  (*n* 151) | | SSBs tax condition  (*n* 126) | | Nutrient profiling tax condition (*n* 109) | |
| --- | --- | --- | --- | --- | --- | --- | --- | --- |
|  | *n* or mean | % or SD | *n* or mean | % or SD | *n* or mean | % or SD | *n* or mean | % or SD |
| Age (years), mean and SD | 48.4 | 15.7 | 48.5 | 16.3 | 48.6 | 15.3 | 48.2 | 15.5 |
| Sex, n and % |  |  |  |  |  |  |  |  |
| Female | 210 | 54.4 | 78 | 51.7 | 77 | 61.1 | 55 | 50.5 |
| Male | 176 | 45.6 | 73 | 48.3 | 49 | 38.9 | 54 | 49.5 |
| Educational level, n and % |  |  |  |  |  |  |  |  |
| Low | 64 | 16.6 | 20 | 13.2 | 19 | 15.1 | 25 | 22.9 |
| Moderate | 141 | 36.5 | 44 | 29.1 | 56 | 44.4 | 41 | 37.6 |
| High | 181 | 46.9 | 87 | 57.6 | 51 | 40.5 | 43 | 39.4 |
| BMI (kg/m^2^), mean and SD |  | 5.8 | 27.5 | 6.0 | 26.5 | 5.7 | 26.0 | 5.4 |
| Weight status, n and % |  |  |  |  |  |  |  |  |
| BMI < 25 kg/m^2^ | 178 | 46.1 | 65 | 43.0 | 61 | 48.4 | 52 | 47.7 |
| Overweight | 128 | 33.2 | 49 | 32.5 | 38 | 30.2 | 41 | 37.6 |
| Obese | 80 | 20.7 | 37 | 24.5 | 27 | 21.4 | 16 | 14.7 |
| Household size, mean and SD | 2.3 | 1.2 | 2.3 | 1.2 | 2.4 | 1.3 | 2.4 | 1.2 |
| Household composition, mean and SD |  |  |  |  |  |  |  |  |
| % of household 14 years or older | 91.7 | 17.9 | 91.4 | 18.6 | 91.2 | 18.3 | 92.7 | 16.6 |
| Household monthly income (gross in €)^a^, n and % |  |  |  |  |  |  |  |  |
| Low (0 – 2000) | 103 | 26.7 | 38 | 25.2 | 33 | 26.2 | 32 | 29.4 |
| Medium (2000 – 3000) | 96 | 24.9 | 37 | 24.5 | 31 | 24.6 | 28 | 25.7 |
| High (3000+) | 187 | 48.4 | 76 | 50.3 | 62 | 49.2 | 49 | 45.0 |
| *Potential modifying variables* | | | | | | | | |
| **STRESS** |  |  |  |  |  |  |  |  |
| Perceived stress^b^, mean and SD | 2.1 | 0.7 | 2.1 | 0.7 | 2.2 | 0.8 | 2.1 | 0.8 |
| Perceived stress, n and % |  |  |  |  |  |  |  |  |
| Unable to control important things in life |  |  |  |  |  |  |  |  |
| Never | 111 | 28.8 | 39 | 25.8 | 35 | 27.8 | 37 | 33.9 |
| Almost never | 115 | 29.8 | 45 | 29.8 | 45 | 35.7 | 25 | 22.9 |
| Sometimes | 95 | 24.6 | 43 | 28.5 | 20 | 15.9 | 32 | 29.4 |
| Often | 48 | 12.4 | 20 | 13.2 | 19 | 15.1 | 9 | 8.3 |
| Always | 17 | 4.4 | 4 | 2.6 | 7 | 5.6 | 6 | 5.5 |
| Difficulties piling up so high that they could not be overcome |  |  |  |  |  |  |  |  |
| Never | 200 | 51.8 | 74 | 49.0 | 62 | 49.2 | 64 | 58.7 |
| Almost never | 104 | 26.9 | 45 | 29.8 | 39 | 31.0 | 20 | 18.3 |
| Sometimes | 63 | 16.3 | 28 | 18.5 | 16 | 12.7 | 19 | 17.4 |
| Often | 17 | 4.4 | 3 | 2.0 | 9 | 7.1 | 5 | 4.6 |
| Always | 2 | 0.5 | 1 | 0.7 | 0 | 0.0 | 1 | 0.9 |
| Unable to handle personal problems |  |  |  |  |  |  |  |  |
| Never | 122 | 31.6 | 49 | 32.5 | 38 | 30.2 | 35 | 32.1 |
| Almost never | 142 | 36.8 | 53 | 35.1 | 49 | 38.9 | 40 | 36.7 |
| Sometimes | 81 | 21.0 | 36 | 23.8 | 26 | 20.6 | 19 | 17.4 |
| Often | 18 | 4.7 | 7 | 4.6 | 5 | 4.0 | 6 | 5.5 |
| Always | 23 | 6.0 | 6 | 4.0 | 8 | 6.3 | 9 | 8.3 |
| Feeling that things were not going the way wanted |  |  |  |  |  |  |  |  |
| Never | 45 | 11.7 | 14 | 9.3 | 16 | 12.7 | 15 | 13.8 |
| Almost never | 212 | 54.9 | 88 | 58.3 | 69 | 54.8 | 55 | 50.5 |
| Sometimes | 93 | 24.1 | 35 | 23.2 | 29 | 23.0 | 29 | 26.6 |
| Often | 28 | 7.3 | 12 | 7.9 | 8 | 6.3 | 8 | 7.3 |
| Always | 8 | 2.1 | 2 | 1.3 | 4 | 3.2 | 2 | 1.8 |
| **FINANCIAL CONSTRAINTS** |  |  |  |  |  |  |  |  |
| Financial constraints^c^, mean and SD | 1.8 | 0.8 | 1.8 | 0.8 | 1.8 | 0.8 | 1.9 | 0.9 |
| Financial constraints, n and % |  |  |  |  |  |  |  |  |
| No financial constraints | 159 | 41.2 | 61 | 40.4 | 59 | 46.8 | 39 | 35.8 |
| Low level of financial constraint | 147 | 38.1 | 58 | 38.4 | 42 | 33.3 | 47 | 43.1 |
| Moderate/high level of financial constraint | 80 | 20.7 | 32 | 21.2 | 25 | 19.8 | 23 | 21.1 |
| **Consumer food purchases** |  |  |  |  |  |  |  |  |
| SSB (litres), median and IQR | 1.0 | 3.0 | 1.5 | 3.0 | 0.8 | 3.0 | 0.8 | 2.4 |
| SSB, n and % |  |  |  |  |  |  |  |  |
| 0 litres | 171 | 44.3 | 58 | 38.4 | 60 | 47.6 | 53 | 48.6 |
| 0.75-1.5 litres | 29 | 7.5 | 10 | 6.6 | 9 | 7.1 | 10 | 9.2 |
| 1.5-3 litres | 83 | 21.5 | 39 | 25.8 | 23 | 18.3 | 21 | 19.3 |
| 3-6 litres | 74 | 19.2 | 30 | 19.9 | 26 | 20.6 | 18 | 16.5 |
| 6 litres or more | 29 | 7.5 | 14 | 9.3 | 8 | 6.3 | 7 | 6.4 |
| Proportion healthy (%), mean and SD | 71.5 | 10.9 | 70.8 | 10.2 | 71.6 | 11.1 | 72.4 | 11.6 |
| Total energy (kcal), mean and SD | 32,080 | 17,074 | 32,422 | 16,540 | 32,926 | 18,665 | 30,630 | 15,905 |
| \| ^a^ The median gross monthly income in the Netherlands (2018) is €4450.^1^  ^b^ Measured by four items: (1) “In the last four weeks, how often have you felt that you were unable to control the important things in your life?”; (2) “In the last four weeks, how often have you felt confident about your ability to handle your personal problems?”; (3) “In the last four weeks, how often have you felt that things were going your way?” and (4) “In the last month, how often have you felt difficulties were piling up so high that you could not overcome them?”, indicated on a five-point Likert scale from 1 “always” to 5 “never”. The items (1) and (4) were reverse coded, and based on the four items, a mean score was calculated, resulting in a total continuous score that ranged from one to five, with higher scores representing higher experienced stress.  ^c^ Measured by one item “In the last 12 months, did you have difficulties making end meets on your household income?” indicated on a four-point Likert scale: 1 “No, no difficulties at all”, 2 “No, no difficulties, but I have to pay attention to my expenses”, 3”Yes, a bit difficult, and 4 “Yes, very difficult”. These last two categories were recoded into one category, resulting in 3 levels of financial constraint: 1 “No financial constraint”, 2 “Low level of financial constraint” and 3 “Moderate/high level of financial constraint”. \| \| --- \| \|  \| | | | | | | | | |

^1^ Centraal Bureau voor de Statistiek (2020) Inkomen van Huishoudens; Inkomensklassen,Huishoudenskenmerken. <https://opendata.cbs.nl/statline/#/CBS/nl/dataset/83932NED/table?ts=1597752840176> (accessed March 2021).
